# Low rates of patients meeting return to sport criteria 9 months after anterior cruciate ligament reconstruction: a prospective longitudinal study

**DOI:** 10.1007/s00167-018-4916-4

**Published:** 2018-03-24

**Authors:** Wouter Welling, Anne Benjaminse, Romain Seil, Koen Lemmink, Stefano Zaffagnini, Alli Gokeler

**Affiliations:** 1 Medisch Centrum Zuid, Sportlaan 2-1, 9728 PH Groningen, The Netherlands; 2 Center for Human Movement Science, University of Groningen, University Medical Center Groningen, Antonius Deusinglaan 1, 9713 AV Groningen, The Netherlands; 3 School of Sport Studies, Hanze University Groningen, Zernikeplein 17, 9747 AS Groningen, The Netherlands; 4 Département de l’Appareil Locomoteur, Centre Hospitalier de Luxemburg, 4 Rue Nicolas Ernest Barblé, 1210 Luxembourg, Luxembourg; 5 Sports Medicine Research Laboratory, Luxembourg Institute of Health, 4 Rue Nicolas Ernest Barblé, 1210 Luxembourg, Luxembourg; 6 Rizzoli Orthopaedic Institute, University of Bologna, Via Gulio Cesare Pupilli 1, 40135 Bologna, Italy

**Keywords:** Anterior cruciate ligament, Return to sport, Strength, Hop tests, Movement analysis

## Abstract

**Purpose:**

The purpose of the current prospective study was to assess the changes over time in patients tested at 6 months and 9 months after anterior cruciate ligament reconstruction (ACLR) with a return to sport (RTS) test battery. It was hypothesized that more patients passed RTS criteria at 9 months compared to 6 months.

**Methods:**

Sixty-two ACLR patients performed a test battery at an average of 6.5 ± 0.7 and 9.5 ± 0.9 months after ACLR. All patients underwent a standardized rehabilitation protocol. The test battery consisted of the following tests: a jump-landing task assessed with the Landing Error Scoring System (LESS), three single-leg hop tasks (single-leg hop test, triple-leg hop test, side hop test), isokinetic quadriceps and hamstring strength at 60, 180 and 300°/s and two questionnaires (IKDC and ACL–RSI). Cut off criteria were set as Limb Symmetry Index (LSI) > 90% (for isokinetic strength and for single-leg hop tasks), LESS < 5, IKDC score within 15th percentage of healthy subjects and ACL–RSI > 56 respectively.

**Results:**

At 6 months, two patients (3.2%) passed all criteria. At 9 months, seven patients (11.3%) passed all criteria. Patients improved in all RTS criteria over time except for the IKDC score. Twenty-nine patients (46.8%) did not pass the strength criterion at 60°/s at 9 months after ACLR.

**Conclusions:**

The percentages of patients passing all RTS criteria were low at both 6 and 9 months after ACLR. Quadriceps strength revealed persistent deficits and the lack of improvement in the IKDC score questionnaires shows insufficient self-reported knee function for RTS.

**Level of evidence:**

III.

## Introduction

The aim for most athletes who undergo an anterior cruciate ligament reconstruction (ACLR) is to restore full knee stability and functional capacity allowing them to return to sport (RTS) [14, 15]. The decision for RTS after ACLR is one of the most challenging and difficult decisions for clinicians to make [45]. The patient expectations for RTS are high, since 94% expect to return to pre-injury level of sports [16]. However, current RTS rates to competitive sports are relatively low (55%) [2, 5] and the overall re-injury rate after ACLR ranges between 15 and 23% for young athletes (< 25 years) [43]. One major reason for these percentages may be the failure to obtain sufficient knee function and muscle strength, which are suggested to be critical for RTS [36, 37].

Traditionally, RTS is recommended after 6 months. However, this timeframe has been questioned in the literature [2, 14, 18], since the risk of sustaining an ACL re-injury is the highest during the early period of RTS (6–12 months) [21, 26]. To decrease the re-injury risk, it is advised to delay RTS to at least 9 months after ACLR. The most commonly used assessments described in literature for RTS decision making after ACLR are strength testing, performance-based functional testing (e.g. hop tests) and self-reported knee function [39]. For strength tests and hop tests, limb symmetry index (LSI) values are commonly used to calculate the difference in score between the non-injured and injured leg. LSIs of > 90% are commonly considered as cut off values for RTS [1, 20, 31].

RTS after ACLR is complex in nature, and it is suggested to use multifactorial test batteries to determine to readiness for RTS of an athlete [18]. It is unclear how ACLR patients progress on multifactorial RTS criteria over time. Therefore, the purpose of the current prospective study was to assess the changes over time in patients tested at 6 and 9 months after ACLR with a RTS test battery. It was hypothesized that more patients passed RTS criteria at 9 months compared to 6 months.

## Materials and methods

**Fig. 1:**
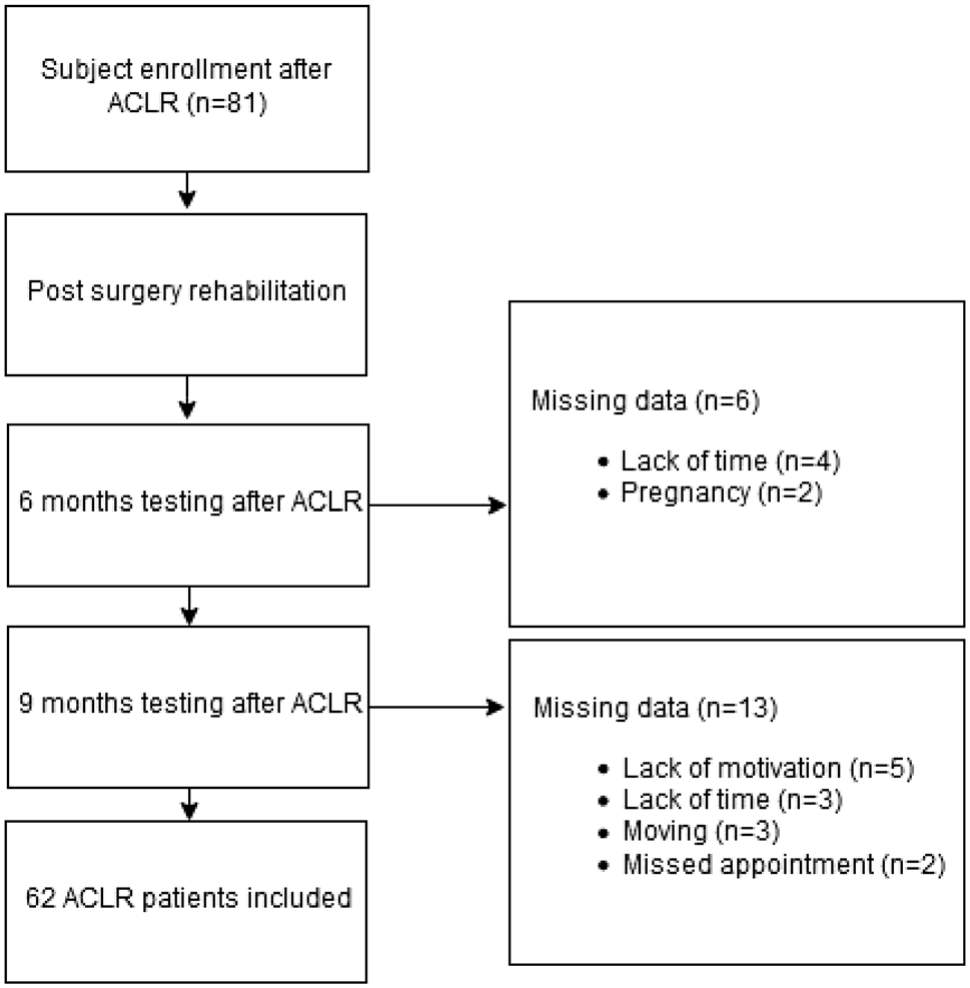
Flow chart of the time line during data analysis. *ACLR* anterior cruciate ligament reconstruction

Patients were prospectively recruited by the same researcher (W.W.) during their rehabilitation in an outpatient physical therapy clinic. Data collection took place between 2015 and 2017. 81 ALCR patients fulfilled inclusion criteria (Fig. 1). Six patients stopped their rehabilitation before 6 months due to a lack of time for rehabilitation (4) or pregnancy (2). Additionally, 13 patients stopped their rehabilitation before 9 months due to lack of motivation (5), a lack of time (3), moving (3) or a missed appointment for the test session (2). Therefore, 62 patients (mean age 24.2 ± 6.2 years) were included. Detailed demographics are presented in Table 1. Inclusion criteria for the patients and the standardized rehabilitation protocol have been described in detail earlier [18]. All patients performed a test battery two times, at an average of 6.5 ± 0.7 and 9.5 ± 0.9 months after ACLR.

**Table 1:** Demographic data included ACLR patients

	All (*n* = 62)	Males (*n* = 45)	Females (*n* = 17)
Age (years)	24.2 ± 6.2	25.3 ± 6.3	21.2 ± 5.0
Mass (kg)	75.8 ± 11.1	78.6 ± 10.3	68.4 ± 10.1
Type of graft (*n*)	HT(36), PT(25), AG(1)	HT(21), PT(23), AG(1)	HT(15), PT(2)
Time post-surgery first-test moment (months)	6.5 ± 0.7	6.5 ± 0.7	6.4 ± 0.6
Time post-surgery second-test moment (months)	9.5 ± 0.9	9.5 ± 0.9	9.4 ± 0.7
Number of therapy sessions first-test moment	52.7 ± 15.6	53.5 ± 12.1	50.7 ± 22.7
Number of therapy sessions second-test moment	74.3 ± 20.1	76.0 ± 18.6	69.7 ± 23.6
Sport	F(45), B(6), H(4), T(3), K(2), R(1), V(1)	F(36), B(5), T(2), K(1), R(1)	F(9), H(4), B(1), K(1), T(1), V(1)

*Kg* kilogram, *HT* hamstring tendon graft, *PT* bone-patellar tendon graft, *AG* allograft, *F* football, *B* basketball, *H* handball, *T* tennis, *K* korfball, *R* rugby, *V* volleyball

### Procedures

All subjects were tested by the same researcher (W.W.). The test battery used in the current study included the following tests in this order [18]: a jump-landing task assessed with the Landing Error Scoring System (LESS), single-leg hop test (SLH), triple-leg hop test (TLH), side hop test (SH), isokinetic strength testing for quadriceps and hamstring strength at a velocity of 60, 180 and 300°/s with 5, 10, 10 maximal concentric repetitions for flexion and extension. After testing, two patient questionnaires were completed by every patient: the International Knee Documentation Committee Subjective Knee Form (IKDC) [27] and the Anterior Cruciate Ligament–Return to Sport after Injury Scale (ACL–RSI) [3].

### Data reduction

All tests used in the test battery have shown to be highly reliable (LESS: ICC = 0.91; SLH: ICC = 0.97; TLH: ICC = 0.80–0.92; SH: ICC = 0.84–0.96; isokinetic device: ICC = 0.91–0.99) [24, 29, 33, 38]. The LESS was analyzed by playing frontal and sagittal videos frame by frame [32]. Muscular strength was tested with an isokinetic device (Biodex System 3; Biodex Medical Systems, Inc, Shirley, NY). For the isokinetic quadriceps and hamstring strength and single-leg hop tasks, LSI values were calculated. Absolute values were normalized to bodyweight (BW) for the isokinetic quadriceps peak torque test at 60°/s for the injured leg. The recommended threshold has been set at > 3.0 Nm/kg [25]. Additionally, hamstring/quadriceps (H/Q) ratios were calculated at 300°/s for the injured leg with a recommended cutoff set at > 55% for females and > 62.5% for males [22]. For the IKDC, the 15th percentile from uninjured athletes was chosen as the cutoff score [27]. Additionally, for the ACL–RSI a cut off score of 56 points was recommended [3]. The study protocol was approved by the Medical Ethical Committee (ID 2012.362) of the University of Groningen, and informed consent was obtained from all patients prior to data collection.

### Statistical analysis

All data were normally distributed as analyzed with SPSS version 20 (SPSS 244 Inc, Chicago, IL). To determine differences between time (6 and 9 months) and legs (non-injured leg and injured leg), a 2 × 2 ANOVA was conducted. Additionally, a 2 × 2 ANOVA was conducted to determine difference between patients with a hamstring tendon graft (HT) and patients with a bone-patellar tendon graft (PT). A power analysis (G*Power, version 3.1.7) was used to calculate the required sample size. With an effect size of 0.50 (medium–large effect ANOVA) and an alpha of 0.05, 27 subjects were required to obtain a power of 0.80 [11].

A regression analysis was used to evaluate whether specific RTS criteria (independent variable) can predict passing/failing the total test battery (dependent variable). Also, the regression analysis was used to evaluate the proportion that each variable can predict the outcome of interest. Participants’ sex and age were used as covariates in the regression analysis. The forward selection method of the regression analysis was used to determine significant predictor variables. Only significant predictors were entered into the regression analysis. Statistical significance was set at *p* < 0.05 level of confidence.

## Results

**Fig. 2:**
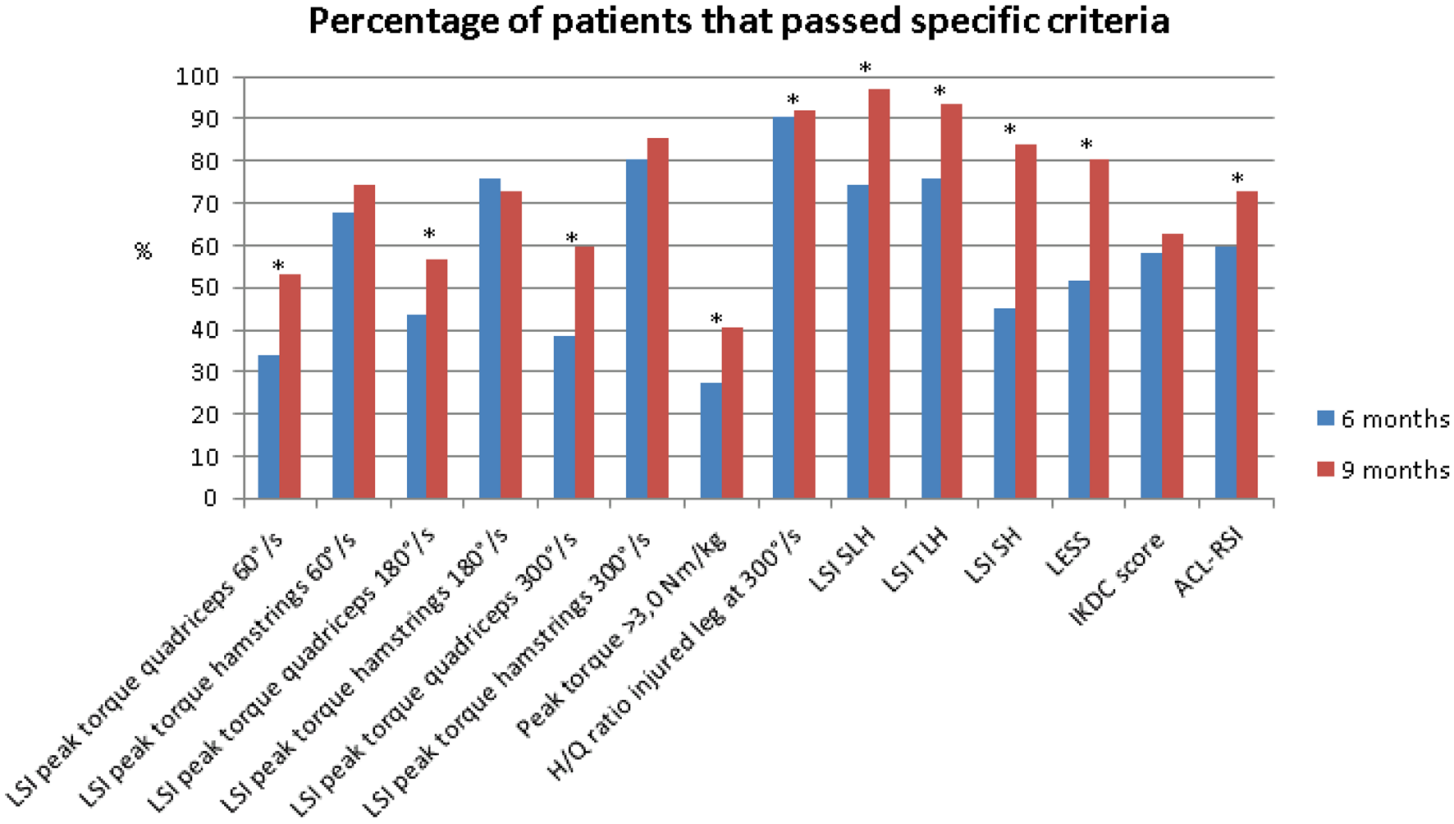
Overview of percentages of patients that passed specific RTS criteria at 6 and 9 months. *LSI* limb symmetry index, °degrees, *s* seconds, *Nm* newton metre, *kg* kilogram, *H/Q* hamstring/quadriceps, *SLH* single-leg hop test, *TLH* triple-leg hop test, *SH* side hop, *LESS* Landing Error Scoring System, *IKDC* International Knee Documentation Committee Subjective Knee Form, *ACL–RSI* Anterior Cruciate Ligament–Return to Sport after Injury Scale, *significant difference

**Fig. 3:**
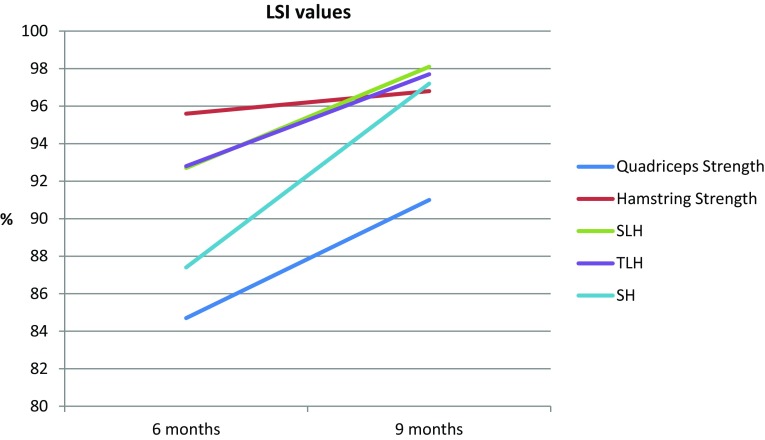
LSI values for males at 6 months and 9 months for quadriceps strength at 60°/s, hamstring strength at 60°/s, single-leg hop test (SLH), triple-leg hop test (TLH) and side hop test (SH). *LSI* limb symmetry index, *SLH* single-leg hop test, *TLH* triple-leg hop test, *SH* side hop test

Of the 62 included patients, 2 patients (3.2%) passed all RTS criteria at 6 months and 7 patients (11.3%) at 9 months. Five patients (8.1%) passed the strength test criteria at 6 months and 13 patients (21.0%) at 9 months. 39 patients (62.9%) passed all hop tests at 6 months and 48 patients (77.4%) at 9 months. An overview of the results can be found in Tables 2, 3 and 4 and Figs. 2 and 3.

**Table 2:** Pass criteria and percentage of patients that passed specific criterion at 6 months and 9 months

Pass criteria and percentage of patients that passed criterion	6 months	9 months
LSI > 90% peak torque quadriceps 60°/s	33.9	53.2
LSI > 90% peak torque hamstrings 60°/s	67.7	74.2
LSI > 90% peak torque quadriceps 180°/s	43.5	56.5
LSI > 90% peak torque hamstrings 180°/s	75.8	72.6
LSI > 90% peak torque quadriceps 300°/s	38.7	59.7
LSI > 90% peak torque hamstrings 300°/s	80.6	85.5
Peak torque > 3.0 Nm/kg for the injured leg at 60°/s normalized to BW	27.4	40.3
H/Q ratio > 55% for females and > 62.5% for males for the injured leg at 300°/s	90.3	91.9
LSI > 90% single-leg hop test	74.2	96.8
LSI > 90% triple-leg hop test	75.8	93.5
LSI > 90% side hop test	45.2	83.9
LESS < 5	51.6	80.6
IKDC score within 15% of healthy gender–age-matched subjects	58.1	62.9
ACL–RSI > 56	59.7	72.6

*LSI* limb symmetry index, ° degrees, *s* seconds, *Nm* newton metre, *kg* kilogram, *H*/*Q* hamstring/quadriceps, *LESS* Landing Error Scoring System, *IKDC* International Knee Documentation Committee Subjective Knee Form, *ACL–RSI* Anterior Cruciate Ligament–Return to Sport after Injury Scale

**Table 3:** Absolute values at 6 months and 9 months, including progress over time

	6 months					9 months					Progress over time	
	Injured leg	Non-injured leg	*p* value	LSI (%)	LSI range (%)	Injured leg	Non-injured leg	*p* value	LSI (%)	LSI range (%)	Injured leg	Non-injured leg
Quadriceps strength 60°/s (Nm)	200.9 ± 49.1	237.3 ± 49.4	< 0.001*	84.7	47.3–105.0	223.9 ± 44.4	246.1 ± 51.1	< 0.001*	91.0	61.2–123.7	< 0.001*	0.016*
Hamstring strength 60°/s (Nm)	127.0 ± 32.1	132.9 ± 31.7	0.001*	95.6	69.6–126.8	134.1 ± 32.1	138.5 ± 31.2	0.021*	96.8	73.4–129.0	< 0.001*	0.001*
Quadriceps strength 180°/s (Nm)	144.8 ± 31.7	167.1 ± 34.7	< 0.001*	86.7	56.1–108.0	159.5 ± 31.2	174.1 ± 35.8	< 0.001*	91.6	73.2–111.0	< 0.001*	< 0.001*
Hamstring strength 180°/s (Nm)	101.8 ± 25.1	106.2 ± 24.6	0.002*	95.9	68.6–120.0	107.3 ± 24.5	110.8 ± 23.9	0.007*	96.8	80.1–131.3	< 0.001*	< 0.001*
Quadriceps strength 300°/s (Nm)	115.0 ± 26.9	131.9 ± 32.7	< 0.001*	87.2	54.6–110.4	122.7 ± 25.1	132.7 ± 27.7	< 0.001*	92.5	73.9–109.3	< 0.001*	0.663
Hamstring strength 300°/s (Nm)	87.2 ± 21.8	89.8 ± 21.2	0.023*	97.1	76.7–130.9	90.4 ± 20.1	92.6 ± 20.0	0.037*	97.6	76.6–132.7	0.031*	0.011*
Single-leg hop test (cm)	150.8 ± 27.3	162.6 ± 25.6	< 0.001*	92.7	61.4–105.8	166.8 ± 23.9	170.1 ± 24.7	0.002*	98.1	88.5–109.3	< 0.001*	< 0.001*
Triple-leg hop test (cm)	477.5 ± 79.1	514.3 ± 78.3	< 0.001*	92.8	69.8–104.4	529.2 ± 77.8	541.4 ± 78.5	< 0.001*	97.7	84.5–112.9	< 0.001*	< 0.001*
Side hop test (times)	45.7 ± 15.8	52.3 ± 13.3	< 0.001*	87.4	47.7–139.3	54.9 ± 13.3	56.5 ± 12.2	0.010*	97.2	74.1–129.3	< 0.001*	< 0.001*
Peak torque normalized to BW (Nm/kg)	2.7 ± 0.6	N.A	N.A	N.A	N.A	3.0 ± 0.6	N.A	N.A	N.A	N.A	< 0.001*	N.A
H/Q ratio (%)	77.1 ± 15.2	N.A	N.A	N.A	N.A	74.7 ± 14.4	N.A	N.A	N.A	N.A	0.028*	N.A
LESS	4.8 ± 2.0	N.A	N.A	N.A	N.A	3.6 ± 1.8	N.A	N.A	N.A	N.A	< 0.001*	N.A
IKDC	81.1 ± 7.8	N.A	N.A	N.A	N.A	81.7 ± 6.9	N.A	N.A	N.A	N.A	n.s	N.A
ACL–RSI	61.7 ± 16.6	N.A	N.A	N.A	N.A	67.3 ± 18.1	N.A	N.A	N.A	N.A	0.009*	N.A

*LSI* limb symmetry index, ° degrees, *s* seconds, *Nm* newton metre, *cm* centimetre, *BW* bodyweight, *H*/*Q* hamstring/quadriceps, *LESS* Landing Error Scoring System, *IKDC* International Knee Documentation Committee Subjective Knee Form, *ACL–RSI* Anterior Cruciate Ligament–Return to Sport after Injury Scale, *N.A*. not applicable

**Table 4:** Combination of specific RTS criteria for the prediction of passing all RTS criteria at 6 and 9 months after ACLR

Dependent variable	Independent variable	*B*	*ß*	*p* value
Passing all RTS criteria at 6 months	Intercept	− 16.360	–	–
*R*² = 0.801	LSI quadriceps strength at 300°/s	0.052	0.179	< 0.001*
	Peak torque > 3.0 N m/kg	1.255	0.268	< 0.001*
	LSI hamstring strength at 180°/s	0.067	0.234	0.002*
	LESS	− 0.317	− 0.217	0.003*
	LSI SH	0.033	0.214	0.004*
	ACL–RSI	0.031	0.179	0.026*
	LSI quadriceps strength at 180°/s	0.076	0.264	0.034*
Passing all RTS criteria at 9 months	Intercept	− 27.062	–	–
*R*² = 0.774	LSI quadriceps strength at 180°/s	0.037	0.118	< 0.001*
	LESS	− 0.462	− 0.298	< 0.001*
	LSI hamstring strength at 180°/s	0.096	0.317	< 0.001*
	IKDC	0.108	0.270	< 0.001*
	ACL–RSI	0.031	0.204	0.022*
	LSI quadriceps strength at 60°/s	0.069	0.263	0.021*
	LSI quadriceps strength at 300°/s	0.097	0.273	0.017*

° degrees, *s* seconds, *Nm* newton metre, *LESS* Landing Error Scoring System, *SH* side hop test, *kg* kilogram, *IKDC* International Knee Documentation Committee Subjective Knee Form, *ACL–RSI* Anterior Cruciate Ligament–Return to Sport after Injury Scale

*Significant predictor

6 months after ACLR, the mean IKDC score was 81.1 ± 7.8 with 36 patients (58.1%) classified as having self-reported knee function with normal ranges and 9 months after ACLR, the mean IKDC score was 81.7 ± 6.9 and 39 patients (62.9%) were classified as having self-reported knee function with normal ranges. For the ACL–RSI, the mean score was 61.7 ± 16.6 at 6 months and 37 patients (59.7%) passed the criteria. At 9 months, the mean ACL–RSI score was 67.3 ± 18.1 and 45 patients (72.6%) passed the criteria.

Significant higher IKDC score (*p* = 0.001) and LSI TLH (*p* = 0.017) were found in patients with a HT graft are compared to patients with a PT graft at 6 months. At 9 months, patients with a HT graft had significant higher LSI quadriceps peak torque at 60°/s (*p* = 0.036) and higher LSI SH (*p *= 0.043) compared to patients with a PT graft.

## Discussion

The main finding of the current study is that only 3.2% of the patients passed all RTS criteria at 6 months after ACLR. Furthermore, only 11.3% of the patients passed all RTS criteria at 9 months after ACLR. The results show a lack of improvement in quadriceps strength and self-reported knee function at 9 months after ACLR. The percentage of patients that passed RTS criteria in the current study after 6 months is similar to the previous results of Gokeler et al. [18]. After 9 months, only 11.3% of the patients in the current study passed the RTS criteria. These findings are in agreement with Toole et al. [39], showed that 13.9% of patients passed RTS criteria (8.2 ± 2.4 months after ACLR).

Almost half of the patients (46.8%) did not pass the quadriceps strength criterion (LSI > 90%) at 60°/s at 9 months after ACLR. Similarly, only 40.3% of the patients passed the criterion > 3.0 Nm/kg for the injured leg at 60°/s. The absolute increase in quadriceps strength between 6 and 9 months was 23.0 Nm, which was lower than the minimal detectable change (MDC) of 33.9 Nm [23]. This indicates a lack of clinical improvement in quadriceps strength from 6 to 9 months after ACLR. Of concern is that these findings are in line with previous research at 6 months after ACLR [18] and, therefore, it is suggested to change the standardized training protocol. Quadriceps strength has been suggested to be essential after ACLR since greater quadriceps muscle strength is a factor associated with successful RTS after ACLR [12]. Furthermore, the results of the current study show that quadriceps strength at 60°/s (only at 9 months after ACLR), 180 and 300°/s (at 6 and 9 months after ACLR) are significant predictors whether patients will pass all RTS criteria. These findings highlight the importance of symmetric quadriceps strength after ACLR.

39 patients (62.9%) passed all hop test criteria at 6 months and 48 patients (77.4%) at 9 months. In addition, patients scored significantly better on all three hop tests at 9 months compared to 6 months after ACLR. The absolute increase in jumping distance between 6 and 9 months on SLH was 10.6% (16.0 cm), which was higher than the MDC of 8.1% [40]. In addition, the absolute increase in jumping distance between 6 and 9 months on the TLH was 10.8% (51.7 cm), which was higher than the MDC of 10.0% [40]. The LSI values of all three hop tests were significantly better at 9 months compared to 6 months after ACLR. The use of LSI is a common method to calculate the score between the injured and non-injured leg [1, 20, 31]. However, caution is warranted for the use of LSI since this method can mask bilateral deficits since the non-injured leg can also be affected by the injury and inactivity time [17]. The use of normative data is suggested to be a more adequate method when analyzing patient data. A comparison with normative data [30] shows that especially our male patients do not meet the jump distance at 9 months after ACLR in the SLH (192.0 cm [30] vs. 175.4 cm for the injured leg) and in the TLH (632.0 cm [30] vs. 558.5 cm for the injured leg). These results show that the use of LSI may underestimate performance deficits and should, therefore, be analyzed with caution when used as a criterion for RTS after ACLR [17, 42].

There is a lack of clinical improvement on the IKDC between 6 and 9 months after ACLR. The absolute change in IKDC score between 6 and 9 months was 0.6, which was lower than the MDC of 8.8 [19]. This indicates insufficient self-reported knee function at 9 months after ACLR. These findings are of concern, since significant lower IKDC scores were found in patients who did not RTS after ACLR [4]. Additionally, the results of the current study show that the ACL–RSI (at 6 and 9 months after ACLR) and the IKDC (at 9 months after ACLR) are significant predictors of passing all RTS criteria in our study. Patients with a PT graft had lower IKDC scores compared to patients with a HT graft at 6 months. Patients did improve their LESS score over time. However, at 9 months after ACLR, still 19.4% of the patients did not pass the LESS < 5 criterion. In more detail, 8.1% of the patients showed a LESS score > 6 (poor jump landing biomechanics) [13, 18, 33]. Furthermore, the LESS is a significant predictor for patients passing all RTS criteria at both 6 and 9 months after ACLR. Therefore, it is recommended to add movement analysis in the decision-making process for RTS [14]. Asymmetrical movement patterns (for example increased knee valgus) are suggested to increase the re-injury risk and should be incorporated in RTS tests [6, 8, 34, 41, 44].

Between graft comparison showed a higher LSI quadriceps strength at 60°/s in patients with a HT graft compared to patients with a PT graft at 9 months. Only 40% of the PT patients passed the LSI > 90% quadriceps strength at 60°/s at 9 months compared to 63.9% of the HT patients. This is in line with previous research, showing a greater quadriceps deficit in PT patients compared with HT patients at 6 months after ACLR [28].

Since only 11.3% of the patients passed all RTS criteria, the results of the current study may suggest that training loads were not high enough during rehabilitation. This could lead to unwanted effects when increasing the training load after returning to the field, since an increase in training load could increase the risk for re-injury dramatically [9]. Therefore, acute/chronic workload ratio (workload last week/workload of the last 4 weeks) should be added in the RTS decision [9]. It is suggested that the acute/chronic workload ratio should be increased carefully during the rehabilitation. In addition, in the last part of the rehabilitation it is suggested to add more sport specific training, for example, field training focused on reactive agility, especially during fatigued circumstances [7, 14]. Especially for RTS (performing at the pre-injury level) or return to performance (performing al least at pre-injury level [14]), fatigue can be a risk factor for re-injury since neuromuscular control is altered under fatigued circumstances [10, 35]. The test battery used in the current study might not be sufficient for the return to performance phase, in which the physical, physiological and psychological demands are much higher compared to RTS.

There are some limitations that should be noticed. The current study gives recommendations in the relevance of the RTS criteria chosen. However, it was not evaluated if the study results pertaining to return to pre-injury level of sports. Prospective studies are needed to determine and evaluate evidence-based RTS criteria. Second, the current study was focused on testing in a closed, clinical environment. Third, in the current study there were dropouts due to a lack of motivation, which could influence the results (attrition bias).

## Conclusion

The percentages of patients passing all RTS criteria were low at both 6 and 9 months after ACLR. The largest improvements were observed in the three hop tests, whilst quadriceps strength revealed persistent deficits. Also, the lack of improvement in the IKDC and ACL–RSI score questionnaires shows insufficient psychological readiness for RTS. Future research should focus on the effects of more progressive quadriceps strength training.
